# Percutaneous sonography-guided treatment of Dupuytren contracture under liquid immersion. Case reports

**DOI:** 10.1016/j.ijscr.2024.110684

**Published:** 2024-11-28

**Authors:** Fabian Moungoundo, Mohamad K. Moussa, Frédéric Schuind

**Affiliations:** a Department of Orthopedics and Traumatology, Université Libre de Bruxelles, Erasme University Hospital, Brussels, Belgium; b Groupe hospitalier Séléstat-Obernai, Séléstat, France

## Abstract

**Introduction:**

Dupuytren's contracture is a fibrotic disorder of the palmar fascia, leading to debilitating finger deformities. Traditional treatments, like open fasciectomy and collagenase injection, carry high risks of complications and recurrence. Ultrasound-guided techniques offer a potentially safer, minimally invasive alternative but are limited by the irregular skin surfaces and flexion deformities in Dupuytren's disease. The Sono-Bath, which immerses the hand in sterile liquid during the ultrasound-guided procedure, improves visualization and facilitates the use of cutting instruments.

**Presentation of the cases:**

Two patients with Dupuytren's contracture were treated using the Sono-Bath. Under local anesthesia, the hand was immersed in sterile saline, allowing for enhanced sonographic imaging. The sterile liquid medium minimized tissue deformation and maintained a clear view of the pathological cords. Using the Sono-Bath, the surgeon was able to precisely position the ultrasound probe at a distance, improving visualization and access to the target area. Percutaneous release of the cords was performed through small punctures, using specialized cutting instruments guided by real-time ultrasound, ensuring accurate and safe cord division.

**Discussion:**

Both cases were successful, achieving full finger extension without complication. The Sono-Bath improved cord visualization and targeting, enabling safer and more effective procedures. Patients reported minimal discomfort.

**Conclusion:**

The Sono-Bath offers a novel, safer approach for minimally invasive treatment of Dupuytren's contracture, enhancing visualization during ultrasound-guided procedures. It also shows potential for broader applications in hand surgery, such as foreign body removal and abscess drainage.

## Highlights

• The study introduces a novel device, the Sono-Bath®, which enables percutaneous, sonography-guided treatment of Dupuytren's contracture under liquid immersion, enhancing visualization and facilitating precise surgical intervention.
• Liquid immersion allows for better sonographic imaging by minimizing tissue deformation, improving the visibility of pathological cords, and making it easier to identify safe entry points for instruments.
• The Sono-Bath® offers a minimally invasive alternative to traditional surgical techniques, providing a more ergonomic and comfortable setup for both the patient and the surgeon.
• Beyond Dupuytren's contracture, the Sono-Bath® shows potential for other hand surgeries, such as foreign body removal, abscess drainage, and tendon laceration diagnosis, due to its ability to maintain a sterile field and improve sonographic clarity.
• The case reports demonstrate the successful application of the Sono-Bath® in treating Dupuytren's contracture, with patients achieving full recovery of finger extension and experiencing minimal discomfort, highlighting the safety and effectiveness of this technique.

## 1 Introduction

Dupuytren's contracture is a progressive fibrotic disorder affecting the palmar fascia, leading to debilitating flexion deformities of the fingers [1,2]. Globally, its prevalence varies, with a comprehensive meta-analysis estimating it at approximately 8.2 % (95 % CI 5.7–11.7 %) [3]. Traditional treatment modalities, including open fasciectomy, closed aponeurotomy, and collagenase injection, are associated with complications such as finger ischemia, nerve injury, infection, complex regional pain syndrome and high recurrence rates [1,2,[4], [5], [6]].

In recent years, ultrasound-guided procedures have become popular as minimally invasive alternatives for the treatment of various hand conditions such as carpal tunnel and trigger finger syndromes [[7], [8], [9], [10], [11], [12], [13]]. A possible surgical application of sonography is for Dupuytren contracture, to guide percutaneous aponeurotomy [14,15]. Sonography allows then precise per-operative identification of the pathological cord to be released and of the neurovascular structures at risk, to be preserved. This is especially important at the base of the finger, where the digital pedicles may be “spirally” displaced by the pathological tissue [16].

The use of sonography for Dupuytren disease is however challenging. Ultrasonography requires direct contact between the probe and the skin, as air disrupts the transmission of ultrasound waves [17,18]. Traditionally, contact gel is used to bridge the gap between the probe and skin, but irregular hard and thickened skin surfaces, such as those seen in Dupuytren's contracture with subcutaneous nodules and palmar skin pits, often necessitate large amounts of gel, which easily flows away from the targeted area. With marked finger flexion contracture, ultrasonography becomes very difficult due to the lack of space between the palm and the volar finger skin, preventing proper positioning of the ultrasound probe.

Another difficulty is that the subcutaneous pathological nodules and cords are very superficial, not well seen even with a high frequency probe. Their release may lead to skin effraction [[17], [18], [19], [20]]. In cases with an important cord that causes a high skin elevation, the small surface of skin in contact with the probe makes it difficult to maintain a large field of view in short axis view.

For simply diagnosis, the solution is to use a stand-off pad or to perform the sonography examination in a water bath filled with water or saline [[21], [22], [23], [24]]. The latter solution imposes cleaning the bath between patients, and that the sonography probe is protected if not waterproof. Such solutions are not applicable for sono-guided Dupuytren surgery, needing sterility, as there is to our knowledge no sterile stand-off pad on the market, and, if choosing to operate under immersion, no dedicated water bath exists to contain the sterile liquid while offering ergonomic conditions for the surgeon and comfortable positioning for the patient during the procedure.

In collaboration with a Swiss company (Spirecut AG, Muttenz), the senior authors of this article (FS and FM) have developed a novel device called the Sono-Bath®, designed to enable percutaneous ultrasound-guided hand surgery under liquid immersion. This new class I medical device allows to maintain the sonography probe at some distance of the skin, improving sonography imaging. Using a liquid medium also presents several advantages: it avoids tissue deformation caused by probe pressure, facilitates the determination of the entry points and trajectories for percutaneous instruments, as both the point of entry and the tissue to be cut are visible in the sonographic field, it eliminates the need for sterile ultrasound gel, even if the skin is very irregular and the finger markedly flexed, and it avoids pain related to the pressure of the probe [23,24]. The probe can also be inclined for better imaging of cords not parallel to the probe.

This article explains, through the presentation of two case reports, the practical use of the Sono-Bath for the percutaneous sono-guided closed aponeurotomy treatment of Dupuytren condition.

## 2 Case presentation (Fig. 1)

Written informed consent was obtained from the patient for publication of this case report and accompanying images. A copy of the written consent is available for review by the Editor-in-Chief of this journal on request.

**Fig. 1:**
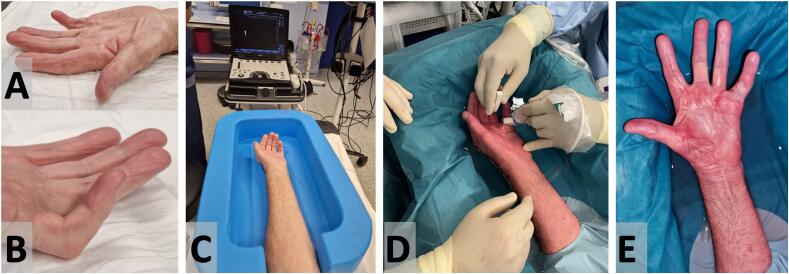
: Dupuytren contracture of case 1, affecting the little finger. Fig. 1-A,B: pre-operative appearance. Fig. 1-C: installation of the hand in the Sono-Bath, on a side table, with at the end of the side table the sonograph, before disinfection. Fig. 1-D: percutaneous chord release using a trigger-finger Sono-Instrument under immersion. Fig. 1-E: aspect of the hand at the end of the operation, before confection of the dressing.

This case was reported in line with the SCARE criteria [25].

### 2.1 History and physical examination

This 76-year-old retired right-handed patient presented with bilateral Dupuytren's contracture. The patient also suffers lymphoid leukemia and had previously undergone bilateral total hip replacements. On the left side, the Dupuytren's contracture caused a flexion deformity of the little finger and painful triggering of the ring finger due to pathological thickening of the A0 and A1 annular pulleys. Initially, the patient sought treatment only for this trigger finger, which was successfully addressed in a first operation using percutaneous ultrasound-guided technique. One year later, the patient requested treatment of the Dupuytren contracture of the neighbouring little finger.

Upon physical examination, a palmo-digital cord was causing flexion of the finger, inserting on the ulnar side of the middle phalanx. The extension deficits were 10° at the MCP joint and 84° at the PIP joint, with a normal DIP joint and full finger flexion (Tubiana-Michon stage III – Fig. 1A,B). The patient requested percutaneous treatment similar to the one successful for trigger finger. The patient was scheduled for surgical treatment by closed aponeurotomy at several levels of the cord under hand immersion and local anesthesia.

#### 2.1.1 Surgical positioning and installation

The patient was comfortably installed with his upper extremity on a side table. At first, before disinfection, a sonography was performed, allowing to identify the subcutaneous cord and the digital bundles. There was no spiral chord. Later, the hand of the patient was disposed in the empty Sono-Bath (Fig. 1C). No tourniquet was used. The installation was judged adequate by the surgeon and his assistant, and comfortable by the patient. The upper extremity of the patient was then disinfected till mid-arm. One first sterile upper extremity waterproof operative drape, comprising a tight opening for the arm, was installed, like for any other standard hand surgery. The drape covered the Sono-Bath. A second identical operative drape was disposed over the first one, providing extra-security that the drape arm double opening was totally watertight, to avoid any risk of liquid contamination from the skin of the arm. Both operative drapes, taking the shape of the underlying Sono-Bath, formed the liquid container, and the hand was now disposed in this container. Lukewarm sterile saline was then poured in the container, until the hand was fully immersed at the site to be operated. A sterile cover was disposed around the hockey-stick 8–18 MHz sonography probe, with already some gel on it, avoiding any space containing air between the probe and the sterile transparent cover, as air would block the echoes' transmission. The surgeon held the probe in his left non-dominant hand, at 1-2 cm from the skin, in the liquid surrounding the operated hand; the syringe used to perform the local anesthesia, and the instruments, were hold in his right dominant hand.

To perform the aponeurotomy, it was decided to use a trigger finger Sono-Instrument®, a minimally invasive device designed for surgical release of A1 pulley under sonography guidance during trigger finger surgery. This instrument progressively cuts tense ligament fibers and is relatively ineffective on elastic tissues. Therefore, it is particularly efficient on a Dupuytren cord and relatively safe if touching tendinous and neuro-vascular structures. The instrument has several features to enhance its echogenicity, and comes in a sterile kit, along with a rounded head metallic stylus which could be used to palpate the cord and confirm the completeness of the release.

### 2.2 Surgical technique

The operation started by injecting 2 ml of lidocaine 1 %, superficial and deep to the cord, at mid-palm. Hydrodissection created space for the cutting instrument. A 14-gauge needle was used to make the entry point, allowing the introduction of the trigger finger Sono-Instrument® through this small puncture to progressively cut the cord under sonographic guidance (Fig. 1D). The same procedure was repeated every centimeter distally, up to the proximal phalanx of the finger, and the assistant could feel progressive release of tension along the contracture.

When the cord was cut at five locations, the surgeon applied gentle forceful extension of the finger, breaking the remaining subcutaneous fibers. Full extension was achieved with moderate force (Fig. 1E).

The liquid in the Sono-Bath, moderately turbid from minimal blood oozing from the skin entry points, was then aspirated, and the hand was dried by elevating the arm and using a sponge. A compressive dressing was applied, to prevent the formation of a postoperative hematoma. Upon removing the drapes, the surgeon made sure that the upper arm was dry, confirming that the drapes remained watertight throughout the procedure.

The patient did not complain of any pain during the procedure, except for mild discomfort during the initial anesthetic injection, and suffered no sensation of coldness despite the hand being immersed in liquid for approximately 20 mins. At the end of the operation, there was no skin rupture, and the patient could immediately achieve full active finger motion.

### 2.3 Follow-up

The patient removed his dressing a few hours after the operation. The postoperative course was uneventful, with no pain, no sensory deficit, or any other complication. At the one-month follow-up, the patient had regained almost full range of motion in the finger and was highly satisfied with the outcome. There were no signs of complex regional pain syndrome.

## 3 Case presentation 2 (Fig. 2)

Written informed consent was obtained from the patient for publication of this case report and accompanying images. A copy of the written consent is available for review by the Editor-in-Chief of this journal on request.

**Fig. 2:**
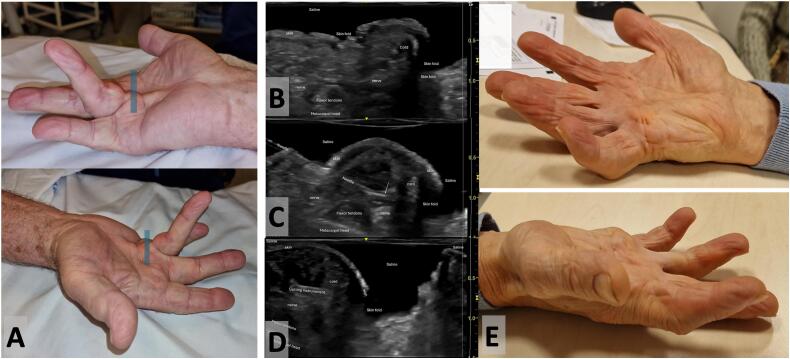
: Dupuytren contracture in the case 2 affecting the ring finger. Fig. 2-A: preoperative condition. The blue rectangle represents the area that was covered by the probe to obtain the sonographic views of Fig. 2-B, 2-C and 2-D. Fig. 2-B: sonographic view under immersion showing on the same image small and large skin folds and bone, tendons, nerves and the Dupuytren cord. Fig. 2-C: targeted injection of local anesthesia around the cord. The white arrow represents the beveled needle tip. Fig. 2- D: sonography guided release of the cord. Fig. 2-E: clinical result, 12 days after the operation. Good recovery of finger extension, no skin rupture. (For interpretation of the references to colour in this figure legend, the reader is referred to the web version of this article.)

This 71-year-old retired right-handed man presented with unilateral Dupuytren's contracture on his right hand. This patient, with a family history of Dupuytren contracture, had no past medical history. Only his ring finger was affected, with a single palmo-digital cord leading a contracture with lack of extension 50° at MCP joint and 20° at PIP joint (Tubiana-Michon stage II – Fig. 2A). The patient was scheduled for surgical treatment under hand immersion and local anesthesia. The same procedure was performed by the same experienced surgeon, but in this case the cord was particularly thick and non depressible, preventing good positioning of the sonography probe, particularly in short axis view used to perform transversal release of the cord. The use of the Sono-Bath was quite helpful to overcome this issue and to increase the operative field of view (Fig. 2B).

During the procedure, the entry point for local anesthesia and the insertion of the cutting instrument were accurately defined under sonography control, avoiding dangerous trajectories (Fig. 2C and D).

Postoperative result was good without any neuro-vascular lesion and with almost completed recovery of MCP and PIP joint extension (Fig. 2E).

## 4 Discussion

Percutaneous sonography-guided closed aponeurotomy treatment of Dupuytren's contracture under liquid immersion using the Sono-Bath is a viable and potentially safer alternative to traditional methods for releasing Dupuytren contractures.

The water-bath technique has previously been employed for ultrasound imaging in challenging cases where direct contact with the transducer would cause patient discomfort or impede image quality [23,24]. Blaivas et al. utilized a non-sterile bedpan to enhance the visualization of painful superficial pathologies in emergency settings, such as abscesses and foreign body removal, allowing for improved patient tolerance and superior imaging without direct contact [23]. Similarly, Krishnamurthy et al. demonstrated the efficacy of water-bath sonography in pediatric patients with complex extremity pathologies, where it provided superior visualization of superficial structures compared to traditional techniques [24].

The use of the new sterile Sono-Bath is highly relevant for Dupuytren's contracture, where treatment remains controversial due to the high rate of postoperative recurrence [1,2,[4], [5], [6]]. Studies have shown that recurrence rates following complete open fasciectomy can range from 12 % to 32 % [5]. This challenge has led to the development of minimally invasive techniques, such as needle aponeurotomy and collagenase injection, which mechanically or chemically section the pathological cord, respectively [1,[4], [5], [6]]. While these methods initially gained popularity for their minimally invasive nature, subsequent studies revealed significant drawbacks. Needle aponeurotomy has shown quite high recurrence rates, reaching up to 55 %, while collagenase injections were associated with skin tears and other complications [5]. Both techniques are performed blindly, posing a risk to neurovascular structures, particularly at the base of the finger. Additionally, collagenase is no longer available in many countries. A recent systematic review of randomized controlled trials conducted by Soreide et al. concluded that there is insufficient evidence to definitively establish the superiority of one technique over another [1].

In an effort to improve the precision and safety of closed aponeurotomy, sonography-guided sectioning of the pathological cord has been recommended [14]. The use of the Sono-Bath facilitates this process by enhancing sonographic visualization of the cord. This is achieved by placing some distance between the tissue and the ultrasound probe, reducing tissue deformation, and enabling more precise targeting of the instrument to the pathological tissue. These features likely contribute to the increased safety of the procedure. As observed in the first reported case, the liquid in the Sono-Bath may become turbid during the procedure due to minor bleeding from the skin entry points, which can make it difficult to locate the needle insertion sites. In such cases, briefly lifting the hand from the liquid or performing a sterile aspiration to replace the turbid liquid with transparent saline can resolve the issue.

In the reported patients, we have used off-label a trigger finger Sono-Instrument. This medical device is indicated for trigger finger treatment [9,10]. Until further clinical studies are conducted to validate its safety and efficacy in Dupuytren's disease, we do not recommend its routine use for this new indication, though it proved to be quite efficient in the present cases. The two reported cases demonstrate the advantages of the new method in terms of safety and precision. Recurrence of the disease has not been assessed in these patients with short follow-up duration. The risk of recurrence is not related to the use of the Sono-Bath, but to the nature of the disease and the choice to treat it by aponeurotomy. Potential complications associated with the Sono-Bath are primarily those of percutaneous release procedures, such as neurovascular injury or incomplete release. Issues like infection, skin tears, or hematoma formation are not directly linked to the Sono-Bath itself.

There are other potential indications of use of the Sono-Bath in hand surgery, in particular for the percutaneous removal of foreign bodies, where precise targeting is essential. The use of the Sono-Bath is also helpful for sonography exploration in case of an open wound when the use of gel is contra-indicated, and in the case of marked pain due to an underlying abcess or bone fracture, when the patient cannot tolerate the pressure of the probe exerted by the physician. For the diagnosis of tendon laceration, the use of the Sono-Bath may facilitate the diagnosis, as the probe can be inclined, allowing better distinction between tendon laceration and anisotropy artifact. In other applications, liquid immersion in the Sono-Bath may help prevent air from entering the operative field during surgical manipulations under sonography, which could block ultrasound echoes and distort visibility.

In conclusion, the authors believe that the Sono-Bath may facilitate percutaneous ultrasonography-guided treatment of Dupuytren disease using closed aponeurotomy at several levels. The technique is obviously contra-indicated when operating a recurrency. Future research should consist in randomized controlled trials to compare sonography-guided closed aponeurotomy with Sono-Bath with traditional techniques, evaluating quantitatively long-term outcomes such as recurrence rates and functional improvement. The Sono-Bath may have other applications in hand surgery.

## Author contribution

**Fabian Moungoundo MD, PhD**: Conceptualization, methodology, data collection, and critical revision of the manuscript, and approval of the final version for submission.

**Mohamad K Moussa MD, MSc**: Methodology, data collection, manuscript drafting, and critical revision of the manuscript.

**Frédéric Schuind MD, PhD**: Conceptualization, supervision, project administration, critical review and editing of the manuscript, and approval of the final version for submission.

## Consent

Written informed consent was obtained from the patients for publication of this case report and any accompanying images. Copies of the written consents are available for review by the Editor-in-Chief of this journal upon request.

## Provenance and peer review

Not commissioned; externally peer reviewed.

## Ethical approval

Ethical approval was not required for these two case reports as it is a retrospective analysis of anonymized clinical data and written informed consent was obtained from the patients for publication.

## Patient and public involvement

Patients and/or the public were not involved in the design, or conduct, or reporting, or dissemination plans of this research. Though feedback about the collection of information for the database is encouraged.

Participant gave informed consent to participate in the study/ collection of data before taking part.

## Guarantor

Prof Fréderic Schuind.

## Sources of funding

This study did not receive any specific grants from funding agencies in the public, commercial, or not-for-profit sectors.

Should this article be accepted, the open access publication fees will be covered by Spirecut. No additional external funding was received for this study.

## Declaration of competing interest

Fabian Moungondo, MD, PhD received royalties from Spirecut.

Frédéric Schuind, MD, PhD holds shares in Spirecut.

Mohamad Moussa declare no conflicts of interest related to this manuscript.

## Data Availability

Data are available upon reasonable request.
